# The structure of *Legionella pneumophila* LegK4 type four secretion system (T4SS) effector reveals a novel dimeric eukaryotic-like kinase

**DOI:** 10.1038/srep14602

**Published:** 2015-09-30

**Authors:** Ali Flayhan, Célia Bergé, Nathalie Baïlo, Patricia Doublet, Richard Bayliss, Laurent Terradot

**Affiliations:** 1 UMR 5086 BMSSI CNRS-Université de Lyon, Institut de Biologie et Chimie des Protéines, 7 Passage du Vercors, F-69367 Lyon Cedex 07, France; 2 Legionella Pathogenesis Group, International Center for Infectiology Research, Université de Lyon Lyon, France; 3 INSERM U1111 Lyon, France; 4 Ecole Normale Suptérieure de Lyon Lyon, France; 5 Centre International de Recherche en Infectiologie, Université Lyon 1 Lyon, France; 6 CNRS, UMR5308 Lyon, France; 7 Department of Biochemistry, University of Leicester, Leicester LE1 9HN, United Kingdom

## Abstract

Bacterial pathogens subvert signalling pathways to promote invasion and/or replication into the host. LegK1-4 proteins are eukaryotic-like serine/threonine kinases that are translocated by the Dot/Icm type IV secretion system (T4SS) of several *Legionella pneumophila* strains. We present the crystal structures of an active fragment of the LegK4 protein in apo and substrate-bound states. The structure of LegK4^1–445^ reveals a eukaryotic-like kinase domain flanked by a novel cap domain and a four-helix bundle. The protein self-assembles through interactions mediated by helices αF and αG that generate a dimeric interface not previously observed in a protein kinase. The helix αG is displaced compared to previous kinase structures, and its role in stabilization of the activation loop is taken on by the dimerisation interface. The apo-form of the protein has an open conformation with a disordered P-loop but a structured activation segment in absence of targeted phosphorylation. The nucleotide-binding site of LegK4 contains an unusual set of residues that mediate non-canonical interactions with AMP-PNP. Nucleotide binding results in limited changes in the active site, suggesting that LegK4 constitutive kinase activity does not depend on phosphorylation of the activation loop but on the stabilizing effects of the dimer.

Protein phosphorylation is one of the most common mechanisms of regulation and signalling^1^. If protein phosphorylation on Ser, Thr, and Tyr residues was for a long time perceived as a eukaryote-specific activity, it is now a well-established signalling mechanism in bacteria^2^ and archaea^3^. Eukaryotic kinases are grouped together in the eukaryotic protein kinase superfamily based on sequence homology between their kinase domains^4^. These domains are typically organized into 12 conserved subdomains that fold in a characteristic two-lobed catalytic core structure, with the catalytic active site lying in a deep cleft formed between the two lobes (reviewed in^5^). The N-terminal lobe is involved primarily in binding and orienting the phospho-donor ATP molecule, whereas the C-terminal lobe binds the protein substrate and initiates the transfer of the phosphate group. The structural basis governing kinase activation has been widely documented (see review^6^). While kinases adopt a variety of conformations in their inactive states, active kinases adopt a single arrangement of conserved structural features. This includes two sets of hydrophobic residues that form spines running through the interior of the kinase which when properly formed, secure the proper positions of catalytic residues^7^. The C-spine contributes to the binding of ATP, and is completed by the adenine base. Formation of the R-spine is coupled to the stabilization of the activation loop, a region that contains one or more sites of activating phosphorylation within most kinases.

Because of its essential role in eukaryotic cells, interfering with host-cell proteins phosphorylation is a common strategy used by bacterial pathogens to hijack cell functions^8^. A growing number of bacterial effectors targeting phosphorylation pathways have been shown to play a role in infection (reviewed in^9^). Among them, a number of effector kinases are found to be injected into host-cell via dedicated machinery including type 3 (T3SS) or type 4 (T4SS) secretion systems. Recent structural studies have shed light onto some of these bacterial effectors kinases^10^. These include NleH1 and NleH2 from entheropathogenic and enterohaemorrhagic *Escherichia coli*^11^, CtkA from *Helicobacter pylori*^12^, OspG from *Shigella*^13^,^14^. Although these proteins belong to the kinase family, they were found to be clearly divergent from a sequence and structural point of view with eukaryotic kinases. In particular, only the motifs I to VII of characteristic 12 motifs are present, and these effectors lack an activation loop as well as helices αG, αH and αI (also named ‘GHI domain’ in^10^).

Other effector kinases seem to have been acquired through gene transfer and can be considered as eukaryotic-like kinases. These include *Salmonella Typhimurium* SteC, *Yersinia pestis* YpkA, and *Legionella pneumophila* LegK kinases family. The later stands out as unique given that four of these proteins are translocated via the Dot/Icm T4SS^15^. *L. pneumophila* is an intracellular Gram-negative bacterium able to infect human alveolar macrophages and to cause Legionnaires’ disease, a severe pneumonia. Instrumental to its survival in infected cells is *L. pneumophila* capacity to re-route the host vesicle trafficking pathways so that it avoids fusion with lysosomes and safely multiplies inside ER-like membrane bound vacuoles (Legionella containing vacuoles or LCVs)^16^. The Dot/Icm T4SS plays a key role in infection and is absolutely required for the intracellular multiplication of the pathogen^17^,^18^. The Dot/Icm system translocates nearly 300 effectors (reviewed in^19^,^20^,^21^), which target various cellular pathways such as anti-apoptosis^22^ and vesicle trafficking^23^, to modify the cell environment in favour of *L. pneumophila* replication.

Sequence analysis of LegK proteins showed that they range from 50 to 100 kDa in mass and share a eukaryotic-like Ser/Thr kinase domain containing the Hanks motifs^15^. While belonging to the same family of effectors, the functions of the LegK proteins are not redundant. On the one hand, LegK1 indirectly regulates the expression of a group of host genes involved in inflammation and anti-apoptosis by inducing the specific activation of the innate immunity pathway NF-κB^24^. The same study demonstrated that LegK1 mimics IκB kinase (IKK) by directly phosphorylating a group of NF-κB inhibitors belonging to the IκB family. On the other hand, LegK2 was found to target a different pathway. Inactivation of LegK2 causes a significant decrease in the virulence of *L. pneumophila* and a delay in the intracellular replication compared to the wild strain^15^. More precisely, LegK2 has been recently shown to phosphorylate the host actin nucleator complex Arp2/3, thus inhibiting actin polymerisation on the LCV and consequently the late endosomes trafficking towards the phagosome. Thus, LegK2 directly contributes to Legionella evasion from the endocytic degradation^25^. Little is known about the functions of LegK3 and LegK4. LegK4 is the largest protein of the family with a large C-terminal domain in addition to the putatively conserved N-terminal functional region containing the kinase domain.

To gain insight into this novel family of T4SS effectors, we have solved the crystal structure of a catalytically active fragment of LegK4 (residues 1–445, LegK4^1–445^) in apo and AMP-PNP bound states. These structures reveal a eukaryotic-like kinase motif and two previously uncharacterized domains. LegK4^1–445^ kinase domain displays unique features, including a modified nucleotide-binding pocket and an original position of the αG helix. A novel mode of dimerisation of the kinase domain was identified in the crystal structures and confirmed by small angle X-ray scattering (SAXS). Our study demonstrates that LegK4^1–445^ is constitutively active and suggests that the dimer assembly stabilizes the active conformation.

## Results and Discussion

### LegK4^1–445^ is active for auto- and substrate phosphorylation

**Figure 1:**
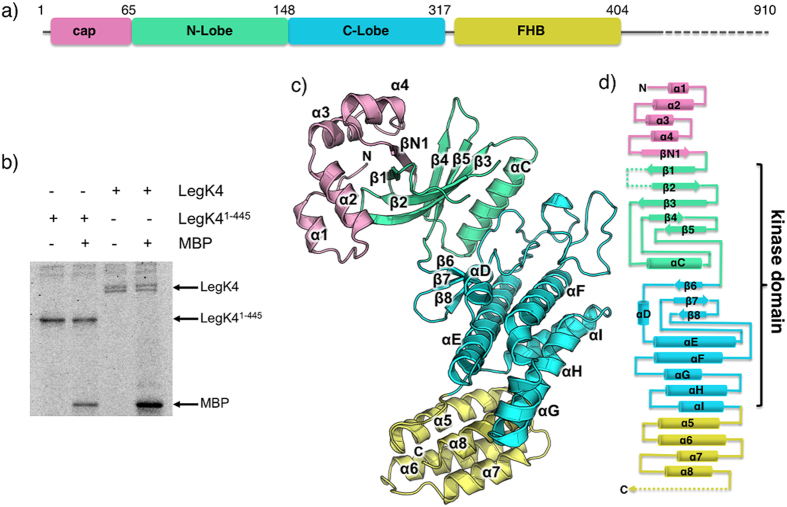
LegK4^1–445^ crystal structure. (**a**) Schematic representation of LegK4^1–445^ protein sequence and the structurally assigned domains of LegK4^1–445^. The “cap” domain (residues 1-65) is represented in pink, the kinase domain is separated in two lobes: the N-lobe (residues 66-148, green) and the C-lobe (residues 149-317, light blue) and the four-helix bundle (FHB) domain (residues 318-406) is coloured in yellow. (**b**) Purified LegK proteins were subjected to *in vitro* auto- and myelin basic protein (MBP) phosphorylation assays in the presence of [γ-32P]ATP. Phosphoproteins were separated by SDS-PAGE and visualised by autoradiography. (**c**) Ribbon representation of the crystal structure of LegK4^1–445^ with domains coloured as in (**a**). (**d**) Topological diagram of LegK4^1–445^, using the same colour code as in panels (**a**,**c**). The missing unstructured regions are represented as dashed lines.

In the process of trying to crystallise LegK4 we observed partial degradation of the full-length protein during purification and identified a stable fragment encompassing residues 1 to 445 (LegK4^1–445^). This fragment corresponds to the most conserved region in the LegK family^15^. We thus generated an expression vector corresponding to this fragment (Fig. 1a) and purified the protein. To determine if LegK4^1–445^ was active, a phosphorylation assay was conducted with either the full length LegK4 or the LegK4^1–445^ fragment. The results show that, as for the wild type, LegK4^1–445^ was able to autophosphorylate using [γ-^32^P]ATP as substrate (Fig. 1b). Moreover, both wild type LegK4 and LegK4^1–445^ were able to phosphorylate the myelin basic protein (MBP) *in vitro* in the presence of [γ-^32^P]ATP (Fig. 1b). Taken together, these experiments indicate that the C-terminal part (residues 446-961) of LegK4 is dispensable for kinase activity.

### Overall structure of LegK4^1–445^

The structure of the apo form of LegK4^1–445^ (apo-LegK4^1–445^) was solved from a SeLegK4^1–445^ crystal and refined at 3.7 Å resolution (*R*_*work*_/*R*_*free*_ of 0.27/0.31) while the structure of substrate-bound LegK4^1–445^ (named hereafter AMP-PNP•LegK4^1–445^) was solved from a crystal grown in the presence of Mg^2+^ and AMP-PNP and refined at 2.5 Å resolution (*R*_*work*_/*R*_*free*_ of 0.20/0.25) (Table 1 and Supplementary Fig. S1). Both apo and substrate-bound crystal forms contain two LegK4^1–445^ molecules in the asymmetric unit (chains A1 and B1 for apo-LegK4^1–445^ and A2 and B2 for AMP-PNP•LegK4^1–445^). The overall root mean square deviation (rmsd) between A1 and B1 is 0.678 Å and 0.460 Å between A2 and B2.

The structure of AMP-PNP•LegK4^1–445^ will be used to describe the general features of the structure of the protein. LegK4^1–445^ is composed of three domains: a small N-terminal domain (residues 1 to 65), followed by the eukaryotic-like kinase domain itself consisting of two lobes, the N and C-lobe (residues 66 to 148 and 149 to 317, respectively) and a C-terminal domain (residues 321 to 406) (Fig. 1a,c,d). No density was observed for residues 407 to 445 suggesting that this part is disordered in the crystal.

**Figure 2:**
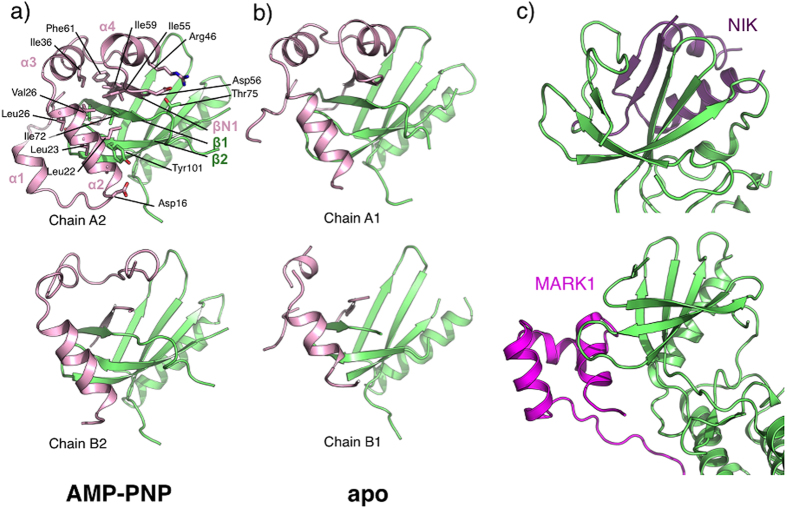
Structural flexibility of the cap domain. Cartoon view of the interface between the cap domain (pink) and the N-lobe (green) of AMP-PNP•LegK4^1–445^ (**a**) and apo-LegK4^1–445^ (**b**). Side chains of principal residues participating in the interface in chain A2 are depicted as ball and sticks with carbon atoms coloured as the cartoon, oxygen in red and nitrogen in blue. (**c**) Structures of mouse NIK (pdb code 4G3C) and human MARK1 (pdb code 2HAK) kinases represented in the same orientation as LegK4 in (**a**) with the N-lobe coloured in green and the NIK N-terminal extension coloured in purple and MARK1 UBA domain coloured in magenta.

The short amino terminal domain, named “cap” hereafter is well defined in chain A1, in which it consists of four small α-helices (α1 to α4) and a β-strand. βN1 runs antiparallel to the N-lobe strands β1 and β4 resulting in an extended six-stranded open β-barrel. The interactions between the cap domain and the N-lobe are extensive, burying a total of 970 Å^2^. A hydrophobic patch formed by α2 Leu22, Leu23 and Val26 and α3 Ile32 and Ile36 interacts with N-lobe residues Ile59, Phe61 (β1), Ile72 (β2), Tyr93 and Leu95 (β3). The interface also involves several hydrogen bonds including salt bridges between Asp56 and Arg46 and between Lys2 and Asp96 (Fig. 2a). Comparison of the cap domain in chains A1, A2, B1 and B2 reveals that parts of the cap domain are disordered as well as the contacting N-lobe elements (Fig. 2a,b). While no obvious homologue structure of this motif could be found using the DALI server, PDBefold^26^ identified several ubiquitin-associated domains (UBA) as remote structural homologues. UBA domains have been described in human AMPK-related kinases, among which are members of the MAP/microtubule affinity-regulating kinases family (MARK)^27^,^28^ (Fig. 2c). In this context, these short helical domains are C-terminal to the kinase domain and can regulate kinase activities through an interaction with the kinase N-lobe at β4 and the β2-β3 loop. Interestingly the cap domain is also similar to the N-terminal extension of the nuclear factor κB-inducing kinase (NIK). Like in LegK4, the extension of NIK also extends the N-lobe β-sheet. The extension of NIK also contains two extra helices, which stabilizes helix αC in the active orientation and maintains the kinase domain in the catalytically competent conformation^29^ (Fig. 2c). Although there are superficial similarities between the cap domain of LegK4 and UBA domains of MARK family kinases and the N-terminal extension of NIK, they bind to different faces of the N-lobe (Fig. 2a–c).

A structure-based sequence alignment of the LegK proteins suggests that the cap domain might be conserved in LegK1 and possibly in LegK2 but not in LegK3, which has a much shorter sequence (Fig. S1). The role of this domain is at present unclear in LegK4 but a LegK1 mutant deleted of the corresponding domain was inactive *in vivo*^24^. It is therefore likely that the cap domain fulfils a structural role in stabilizing the kinase N-lobe, similar to what has been previously observed with N-terminal extensions to kinase domains, such as the NIK^29^,^30^ or Nek7^31^.

The kinase domain (KD) of LegK4^1–445^ is very similar to other eukaryotic Ser/Thr kinases displaying the canonical N and C-lobes. The N-lobe contains a six-stranded antiparallel open β-barrel (βN1 and β1 to 5) and the αC α-helix. The C-lobe is composed of three antiparallel β-strands (β6-8) and six α-helices (αD to I). The KD of LegK4^1–445^ contains the twelve conserved subdomains characteristic of eukaryotic kinases^4^. The KD is flanked by a four-helix bundle (FHB, α5 to α8) with the α5 inserted into a groove formed by αH and the loop αI-α5 (Fig. 1). FHB domains are ubiquitous and have numerous and divergent functions from secreted bacteriocins to human cytoskeleton binding proteins. Noteworthy a FHB domain is present in MAST1 (pdb code 2M9X), a member of the microtubule-associated Ser/Thr-protein kinase family but with no function yet associated. The sequence conservation for the FHB residues is weak amongst the LegK family but secondary structure prediction suggests that LegK1 and LegK2 might each have an all α-helical domain C-terminal to the KD (Supplementary Fig. S2). It is noteworthy that a mutant of LegK1 deleted of the C-term domain was still active for phosphorylation^24^. The position of the FHB in LegK4, *i.e* away from the kinase domain also suggests that it is likely not involved in the kinase activity, as is the case for LegK1.

### LegK4 displays an atypical active kinase domain

**Figure 3:**
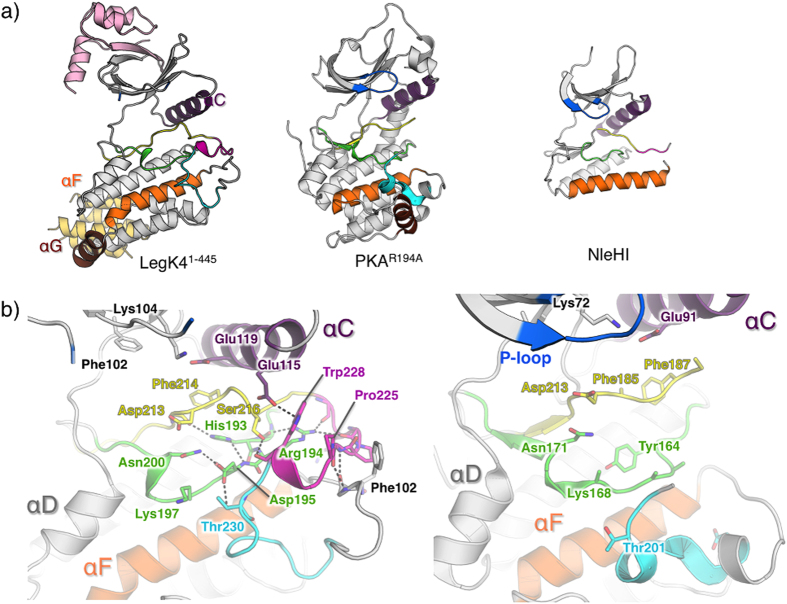
Structure of apo-LegK4^1–445^. (**a**) Cartoon representation of the crystal structures of apo-LegK4^1–445^, mouse PKA mutant R194A (pdb code 4DFY) and NleH1 (pdb code 4LRJ). The helices αC, αF and αG are coloured in purple, orange and brown, respectively. The P- loop is coloured in blue and the activation segment coloured as follow: catalytic loop (green), Magnesium-binding loop (yellow), activation loop (magenta) and P+1 loop (cyan). (**b**) Close-up view of the catalytic site of apo-LegK4^1–445^ (left) and PKA mutant (right) coloured as in (**a**). A side chain of important residues are shown as ball and sticks and coloured as the cartoon (carbon), red (oxygen) and blue (nitrogen). Main hydrogen bonds are indicated as dashed lines.

The KD of LegK4^1–445^ contains all the conserved elements of eukaryotic-like kinases, including an activation loop and a GHI domain contrasting with the minimal KD of the effector kinase so far characterized (Fig. 3a, Supplementary Fig. S2). Moreover, the P+1 loop activation segment residues Gly229 and Thr230 forms the GT motif, characteristic of Ser/Thr kinases^32^. Compared to the eukaryotic kinases, the structure displays however key differences in functional motifs. The position of the αG is distinctive in LegK4^1–445^, as it is distant from the activation loop of the enzyme (Fig. 3a). In its usual position, αG plays an important role by binding to the P+1 loop and providing a scaffold/interface for protein substrate binding^6^. The active site of LegK4^1–445^ displays a disordered P-loop (or glycine-rich loop, residues 79 to 87).

Remarkably, the activation segment of LegK4^1–445^ is ordered (Fig. 3b). In most kinases, the activation segment is disordered in absence of phosphorylation as illustrated by the structure of a PKA mutant (R194A) that does not autophosphorylate on its activation loop^33^ (Fig. 3b). Ordering of the activation segment in many kinases requires phosphorylation of a serine or threonine residue in the activation loop. In the apo-LegK4^1–445^ structure, a dense hydrogen bond network stabilizes the activation segment conformation (Fig. 3b). In particular, the catalytic loop residue Asp195 interacts with Asn200 and with Thr230 (P+1 loop) and His193 side chain binds to the hydroxyl group of Val212 (magnesium binding loop). The activation segment is further stabilized by Arg194 side chain which interacts with the main chain hydroxyl groups of Asp220 and Glu222, thereby connecting the catalytic and activation loops. In contrast to canonical protein kinases such as PKA, in which there is a short helix (αEF) between the APE motif at the C-terminal end of the activation loop and the start of αF, this region of LegK4 adopts an extended loop conformation that forms extensive stabilizing interactions with the activation loop. These include main chain interactions between Pro225 and Leu224 with Phe244 and Ile245. In addition, the position of the αC helix near the active site is stabilized via a hydrogen bond between its residue Glu115 and Trp228 (Fig. 3b).

These observations suggested that the protein might be in an active conformation without phosphorylation of the activation loop. To test this hypothesis, we searched for phosphorylation site(s) in LegK4 and LegK4^1–445^ by liquid chromatography coupled to tandem mass spectrometry (LC-MS/MS). Proteins were incubated with ATP followed by trypsin digestion. Mass spectrometry identified residue Thr823 to be phosphorylated in LegK4 and residues Ser422, Ser426 and Thr433 in LegK4^1–445^ (Table 2). Ser422, Ser426 and Thr433 residues belong to the unstructured region, which could not be observed in the crystal structures. Since these are not phosphorylated in the full length LegK4, it is likely that phosphorylation of these residues is non-specific. Neither in LegK4 nor in LegK4^1–445^ was the activation segment found to be phosphorylated. These results strongly suggest that, in agreement with our structural observation, the kinase activity of LegK4 does not require phosphorylation of the activation segment.

### Nucleotide binding to the active site

**Figure 4:**
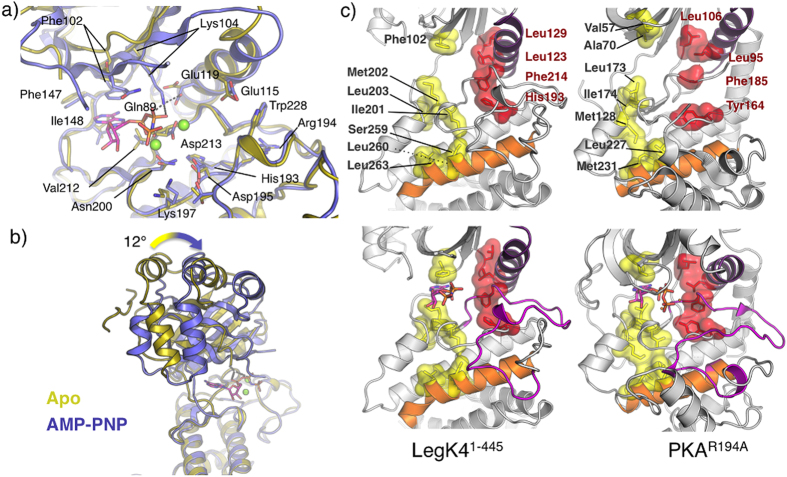
Nucleotide binding to LegK4^1–445^. (**a**) Cartoon representation of superimposed active sites of apo-LegK4^1–445^ (gold) and AMP-PNP•LegK4^1–445^ (blue). Side chains of important residues are shown as ball and stick and coloured as the cartoon (carbon), red (oxygen) and blue (nitrogen). (**b**) Structural superimposition of apo-LegK4^1–445.^(gold) and AMP-PNP•LegK4^1–445^ illustrating the movement of the cap domain and the N-lobe. (**c**) Comparison of the architectures of the C- (yellow surface) and R-spines (red surface) in the apo (upper panels) and nucleotide bound states (lower panels) of LegK4^1–445^ and mouse PKA mutant R194A (pdb code 4DFY).

In the AMP-PNP•LegK4^1–445^ structure, one AMP-PNP molecule and two Mg^2+^ ions were present per LegK4^1–445^ monomer (Fig. 4a). The activation segment, including the catalytic loop, the magnesium binding loop, the activation loop and the P+1 loop, adopts almost the same conformation as in the apo form (overall rmsd = 0.4 Å) (Fig. 4a). The nucleotide binds to LegK4^1–445^ in a canonical manner, between the N- and C-lobe, with the adenine moiety buried in a hydrophobic pocket formed by Phe102, Phe147, Ile148 and Val212. The N1 and N6 amino groups of the adenine are hydrogen bonded to the Ile148 amide and to the Phe147 carbonyl, respectively while the N3 and N7 amino groups interact with surrounding bridging water molecules. Interestingly, the adenine is stacked by the Phe102 side chain. In the vast majority of kinases, this position is occupied by an alanine as part of the consensus “VAIK” sequence. In the case of kinase suppressor of Ras (KSR) 1, mutation of this residue to phenylalanine ablates the binding of ATP^34^ and the pseudokinase RYK, which does not bind nucleotide, also has a phenylalanine at this position^35^. In contrast, the presence of a phenylalanine in this position in LegK4 seems to stabilize the adenine ring rather than preventing nucleotide binding. The two hydroxyls of the ribose moiety are hydrogen bonded to the Asp152 side chain and the Gly199 carbonyl. The α- and β-phosphates interact with the invariant Lys104 and the Gln89, respectively (Fig. 4a). The γ-phosphate does not make direct interaction with the protein. The invariant Asp213, from the magnesium-binding loop, chelates the primary activating Mg^2+^ ion that directly binds the β-phosphate, and bridges the γ-phosphate *via* a water molecule. The secondary Mg^2+^ ion interacts with the Asn200 side chain and bridges the α- and β-phosphates (Fig. 4a).

Comparison of the apo- and AMP-PNP•LegK4^1–445^ structures shows that upon ligand binding, the protein undergoes a significant conformational change. A ∼12° rotation of the N-lobe and the cap domain around the active site brings closer together the two lobes of the catalytic domain as in other kinases (Fig. 4a,b). The P-loop, which usually covers and stabilizes the phosphates of the bound nucleotide, is disordered in apo-LegK4^1–445^, and is still not visible in the electron density maps of the nucleotide-bound structure (Fig. 4a,b). To our knowledge, the structure of LegK4^1–445^ is the first reported structure in which the P-loop is flexible in both, the open and the closed forms of the kinase protein structure. Indeed, the P-loop does not appear to contribute to ATP binding, and other interactions, such as with Gln89 and Lys104, are sufficient to stabilize the position of nucleotide phosphates. It has been suggested that P-loop conformational changes do not necessary reflect a flexible loop but rather rigid movements from one conformation to the other upon substrate binding^36^. The absence of an ordered P-loop suggests that this loop might play either a role in directing the specificity of LegK4 kinase activity, and/or a role in regulation of kinase activity. In either case the P-loop may be stabilized upon interaction with a target protein.

A key difference between the apo- and AMP-PNP•LegK4^1–445^ structures is the αC helix. In the AMP-PNP•LegK4^1–445^ structure, αC Glu119 interacts with the Lys104 from β4 strand, allowing the proper orientation of the AMP-PNP α- and β-phosphates (Fig. 4a). Accordingly, a mutant with the corresponding Lys in LegK1 (Lys121, strain Philadelphia-1) changed to alanine failed to induce NF-κB activation *in vivo*^24^. This interaction is accompanied by a change in αC secondary structure. Upon AMP-PNP binding αC is one turn longer at its N-terminal than in apo-LegK4^1–445^. This rearrangement triggers the inward movement of the N-lobe towards the C-lobe by shortening the β3- αC loop (Fig. 4a,b).

Activation of kinases can also be assessed by the alignment of two sets of spatially conserved hydrophobic residues, termed the catalytic (C-) and regulatory (R-) spines^37^,^38^. In inactive kinases the C and R-spines are disassembled while in active, nucleotide bound kinases, the spines form parallel columns of hydrophobic residues sitting on the αF helix (Fig. 4c). These spines span organize the catalytic infrastructure by restricting inter-lobe movements. As seen in Fig. 4c, the C and R-spines of LegK4^1–445^ are already parallel in the apo form suggesting that the kinase is active. Binding of AMP-PNP completes the C-spine but with minimal structural rearrangement of the column. These observations are in agreement with the constitutive activity of the kinase.

### A novel dimeric form stabilizes essential catalytic elements

**Figure 5:**
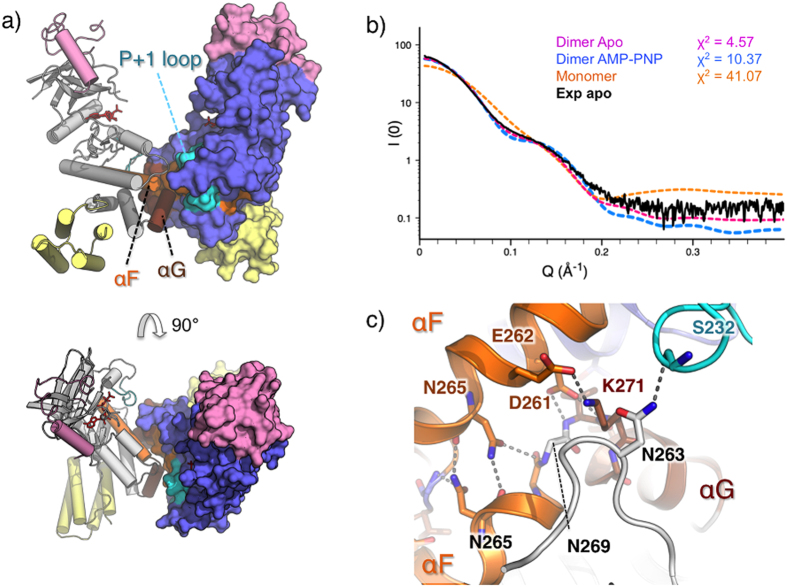
LegK4^1–445^ dimer organisation. (**a**) Side (upper panel) and top (lower panel) views of the dimer with chain A coloured in grey and shown as a surface and chain B coloured in blue and shown as ribbon. The cap and FHB domains of the two chains are coloured in pink and yellow, respectively. (**b**) Comparison of the experimental SAXS curve of apo-LegK4^1–445^ (black) with theoretical SAXS curves of apo-LegK4^1–445^ monomer (orange), dimer (magenta) and AMP-PNP•LegK4^1–445^ dimer (blue). χ2 obtained with FOXS server^51^ are indicated. (**c**) Detailed view of the dimer interface coloured as in (**a**) with participating residues shown as ball and stick.

Both the apo and the substrate bound LegK4^1–445^ crystal forms contain two molecules in the asymmetric unit forming a similar dimer. The interface is symmetric and buries around 900 Å^2^ as determined by the PDBePISA server^39^ (Fig. 5a). To determine if the dimer of LegK4^1–445^ was relevant in solution we used inline size exclusion small angle X-ray scattering (SEC-SAXS)^40^. SAXS data indicates that the Rg of LegK4^1–445^ is 36.1 Å with a Dmax of 111 Å (Fig. 5b, Supplementary Fig. S3). These values are in excellent agreement with the theoretical Rg and Dmax of respectively 34 Å and 111 Å, derived from the dimer observed in crystal structure. The small difference might be due to the disordered region of 45 residues present at the C-terminal portion of each monomer. Comparison of LegK4^1–445^ experimental SAXS data with the theoretical curves obtained with the crystal structures demonstrate that the best fit was obtained with the dimer of apo-LegK4^1–445^ (χ2 = 4.6) compared to the dimer of AMP-PNP•LegK4^1–445^ (χ2 = 10.5) or to the apo-LegK4^1–445^ monomer (χ2 = 41.1) (Fig. 5b). These data clearly show that in solution, LegK4^1–445^ adopts the conformation observed in the crystal structure.

**Figure 6:**
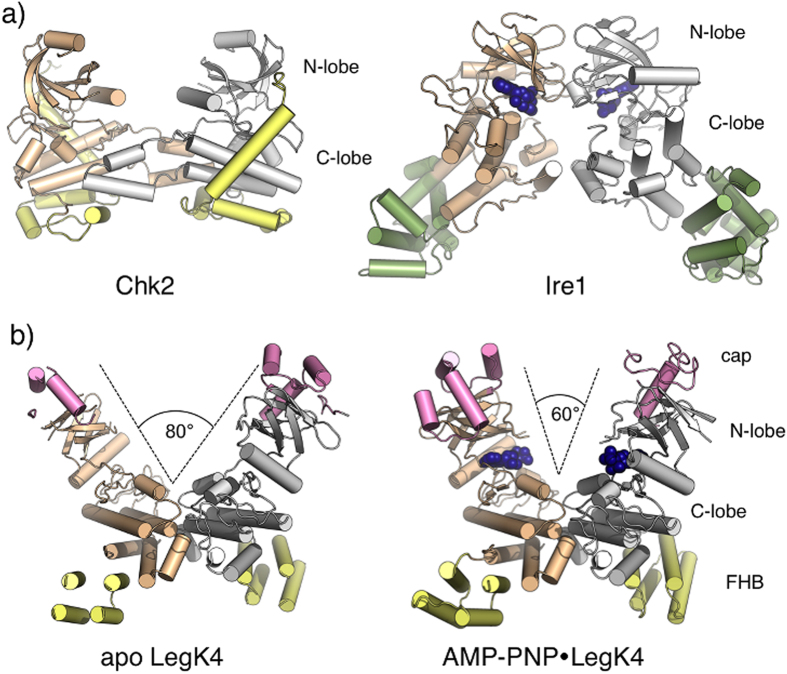
Structural comparison of kinase dimers. (**a**) Cartoon view of the crystal structures of dimeric kinases Chk2 (pdb code 2HK9) and Ire1 (pdb code 3P23). Kinase domains are coloured in wheat and grey and specific domains are coloured in yellow (Chk2) and green (Ire1). AMP-PNP is represented as dark blue spheres in Ire1. (**b**) Cartoon representation of apo- and AMP-PNP•LegK4^1–445^ illustrating the closing of the dimer assembly upon binding AMP-PNP (represented as dark blue spheres). Kinase domains are coloured in wheat and grey, cap domains in pink and FHB domains in yellow.

The dimerisation involves predominantly charged residues from helices αF, αG and the αG-αI loop of the C-lobe and is essentially the same in the apo- and AMP-PNP•LegK4^1–445^ structures (Fig. 5a,c). Briefly, αF Asp261 interacts with the backbone nitrogen of Val270 and Lys271 and Asn265 self interact. Reciprocal salt bridges occur between Glu262 and Lys271 residues. Interestingly, Ser232 located within the P + 1 loop forms a hydrogen bond with Asn163 from the αD-αE loop and this interaction occurs in both apo and substrate-bound forms (Fig. 5c). This stabilization role is usually played by helix αG, which is displaced here. The organisation of the dimer thus suggests that the assembly participates in the ordering of the activation loop in absence of phosphorylation. Dimerisation of kinases can promote activation through trans-phosphorylation or allosteric mechanisms (reviewed in^6^,^41^). Comparison of LegK4^1–445^ dimer with the structures of other kinases that dimerise with the active sites facing each other, such as Chk2^42^ and Ire1^43^, reveals that the dimerisation mode of LegK4 is novel (Fig. 6a). Interestingly, upon binding to AMP-PNP, the dimer interface seems to act as a pivot onto which the two subunits close (Fig. 6d). Given the central part of the dimer obstructs part of the kinase surface that is usually involved in binding protein substrates, this feature might restrict the substrate size. Together with the observation that the P-loop remains disordered in the presence of nucleotide, which is instead stabilized by atypical residues such as Phe102 and Gln89, it is possible that LegK4 binds protein substrates in a different orientation than most kinases.

## Concluding Remarks

Eukaryotic-like kinase effectors represent a common strategy for bacterial pathogens to subvert host cell defences. While bacterial kinase effectors identified so far contained minimal kinase domains^10^, the structure of LegK4 reported here presents the characteristics of eukaryotic regulatory kinases albeit with remarkable, original modifications. Notably, nucleotide binding does not involve the P-loop, which has a highly divergent sequence, but is instead mediated by unusual residues in nearby structural elements. The newly described dimer and atypical activation loop suggest a structural explanation for LegK4 constitutive activity that was unpredictable from sequence analysis. Our analysis indicates that some of these elements might be shared by LegK1 and possibly other members of the family. The structure also points toward specific mechanisms of kinase regulation within the family that could be exploited by the bacteria to re-route different host pathways. While a number of these questions remain open, our study has paved the way for detailed and mechanistic studies aiming at understanding the role of eukaryotic-like kinases during bacterial infection.

### Experimental Procedures

#### Cloning, over-expression and protein purification

PCR was used to amplify the DNA fragment encoding for LegK4 (forward primer 5′-CACCATGAAATTGCCTTCGGTTTCATGAAT-3′, reverse 5′-TTAATATGGCAAAATGATGACGTTGC-3′) and LegK4 amino-acids 1 to 445 (reverse primer 5′-TTAATCTGAAACTTGTTTAATCGCTGC) and inserted into the pET151/D TOPO (Invitrogen) following manufacturer procedure. The DNA fragment coding for LegK_4_ amino-acids 1 to 445 was inserted into the pET151/D- to express the protein LegK4^1–445^ in fusion with a N-terminal His_6_-tag followed by a tobacco etch virus protease (TEV) cleavage site. The resulting vectors were introduced into the *E. coli* BL21 (DE3) and the bacteria were cultured at 37 °C in Lysogeny broth medium supplemented with 100 μg/ml ampicillin up to an optical density at 600 nm of 0.6–0.9. Protein expression was induced by adding 1 mM Isopropyl β-D-1-thiogalactopyranoside and the bacteria were grown at 20 °C for 16 hours. Cells were harvested by centrifugation and flash frozen in liquid nitrogen before being stored at −80 °C. Frozen cells were re-suspended in lysis buffer (50 mM Tris, pH 8.0, 0.5 M NaCl, 10% Glycerol (V/V) and 20 mM Imidazole) supplemented with 5 U/ml of benzonase (Novagen) and a tablet of a Ethylenediamine tetra-acetic acid (EDTA)-Free protease inhibitors cocktail (Roche). Re-suspended cells were disrupted by sonication and centrifuged for 20 min at 16000 g at 4 °C. The supernatant was then loaded onto a HisTrap 5 ml column (GE Healthcare), previously equilibrated with 15 ml of equilibration buffer (20 mM Tris, pH 8.0, 150 mM NaCl, 5% glycerol and 20 mM Imidazole). The column was washed with 20 ml of the equilibration buffer and 20 ml of equilibration buffer containing 50 mM Imidazole. The proteins were eluted with a 50–500 mM Imidazole gradient). The His_6_-tag of LegK4 and LegK4^1–445^ were then cleaved by mixing a His_6_ tagged TEV protease and the fusion protein at 1:40 (w/w) ratio in the presence of 0.5 mM EDTA and 0.5 mM Dithiothreitol (DTT). The mixture was dialysed against 20 mM Tris, pH 8.0, 150 mM NaCl, 5% Glycerol (V/V) at 4 °C. Cleaved LegK4 or LegK4^1–445^ were then separated from the uncleaved fraction and TEV protease by reloading the dialysed protein onto a Nickel affinity column. Both LegK4 and LegK4^1–445^ were then further purified on an anion exchange column (HiTrap Q; 5 ml; GE Healthcare) equilibrated with 20 mM Tris, pH 8.0 and 5% glycerol. The proteins were eluted with a NaCl gradient (0 to 1 M). A final size exclusion chromatography step (HiLoad 16/600 Superdex 200 pg column; GE Healthcare) was performed in 20 mM Tris, pH 8.0, 150 mM NaCl, 5% Glycerol (V/V). Selenomethionine labelled LegK4^1–445^ (SeLegK4^1–445^) was produced following the protocol described in (13) and purified as LegK4^1–445^.

### Crystallization, data collection and processing

LegK4^1–445^ and SeLegK4^1–445^ were concentrated to 8–10 mg/ml using an Amicon Ultra 10-kDa concentrator. The vapour diffusion sitting drop technique was used for crystallization with drops, consisting of 1 μl protein and 1 μl reservoir buffer equilibrated against 500 μl of reservoir solution in 24-well plates (Intelli-plates, ARI) at 292K. Native LegK4^1–445^ was co-crystallized with 1 mM Adenylyl imidodiphosphate (AMP-PNP) and 2 mM MgCl_2_, which were added to the protein 10–20 minutes prior to setting-up crystallization drops. Plate shaped crystals were obtained for LegK4^1–445^ in complex with AMP-PNP, with a reservoir solution consisting of 10% (w/v) polyethylene glycol (PEG) 20000, 20% (w/v) PEG 550 mono-methyl ether, 30 mM MgCl_2_, 30 mM CaCl_2_ and 100 mM Tris/Bicine pH 8.3. Bipyramidal crystals for SeMet derivative LegK4^1–445^ grew with a reservoir solution 12% (w/v) PEG 4000, 20% glycerol, 0.1 M MOPS/HEPES pH 7.5, 5 mM Hexaammine cobalt (III) chloride. Crystals were then flash-cooled, and stored in liquid nitrogen. Data for native LegK4^1–445^ and Se-LegK4^1–445^ were collected at 100K at ID23EH2 (European Synchrotron Radiation Facility) and Proxima 2a (SOLEIL) beamlines, respectively. Reflections were indexed, integrated, and scaled with the XDS program suite^44^. The high-resolution cut-offs were estimated according to CC1/2^45^. Data statistics and parameters are summarized in Table 1. Crystals of Se-LegK4^1–445^ and LegK4^1–445^ belonged to space group P321 and P12_1_1, respectively and contained two molecules per asymmetric unit.

### Structure solution and refinement

A high-redundancy data set was collected on a single Se-LegK4^1–445^ crystal at the K absorption edge wavelength (0.979 Å). The initial heavy-atom sites were located using AutoSol^46^ from the Phenix program suite^47^. Despite the limited resolution of 3.7 Å, the initial phases had a very good figure of merit (FOM) of 0.580, and an excellent experimental electron density map enabled us to manually build an initial model. The resulting model was then used for molecular replacement calculations using the monoclinic data set at 2.49 Å resolution with Phaser^47^, in which two monomers were positioned with a translation function Z-score of 9.7. The resulting electron density map was then subjected to the AutoBuild program, part of the Phenix program suite^47^. An initial model consisting of 526 residues in 12 chains and 269 water molecules was obtained. Both native and derivative models were completed with sessions of manual model building using Coot combined with model refinement using Phenix^47^. Representative pictures of the electron density map are shown in Supplementary Fig. S1. Figures were generated with Pymol^48^. The coordinates and structure factors of the structures of apo-LegK4^1–445^ and AMP-PNP•LegK4^1–445^ have been deposited in the protein data bank with PDB codes 5CLR and 5CKW, respectively.

### Small angle X-ray scattering

SAXS data were recorded on beamline Swing at SOLEIL Synchrotron (Gif sur Yvette, France) at a wavelength of 1.003 Å on a 17 cm × 17 cm low-noise Aviex charge-coupled device detector positioned at a distance of 1800 mm from the sample, with the direct beam off-centered. The useful Q-range was 0.004–0.61 Å^−1^, where Q = 4πsinθ/λ is the scattering vector, and 2θ is the scattering angle. 50 μl of LegK4^1–445^ (10 mg.ml^−1^) in buffer S (50 mM Tris pH 8.0, 200 mM NaCl, Glycerol 5% (v/v), 1 mM DTT) were injected into a size-exclusion column (Agilent BioSec-3) using an Agilent© HPLC system cooled at 15 °C and eluted directly into the SAXS flow through capillary cell^40^. SAXS data were collected online throughout the whole elution time, with a frame duration of 2 s and a dead time between frames of 1 s. A first data set of 90 frames, collected before the void volume, were averaged to account for buffer scattering. A second data set was collected for the sample, from which the 10 frames corresponding to the top of the elution peak were averaged and used for data processing after baseline subtraction. Data were processed using the local application FOXTROT^49^ and analysed using PRIMUS^50^. Theoretical curves from the models were generated by FoXS^51^.

### *In vitro* phosphorylation assay

*In vitro* phosphorylation of about 2 μg of purified recombinant protein was performed for 30 min at 37 °C in 20 μl of a buffer containing 25 mM Tris-HCl (pH 7.5), 5 mM MgCl_2_, 1 mM DTT, 10 μM ATP, and 5 μCi of [γ-32P]ATP (Perkin-Elmer, Courtaboeuf, France). In some assays, MgCl_2_ was replaced with MnCl_2_. In eukaryotic substrate phosphorylation assays, 1 μg of MBP was added. In each case, the reaction was stopped by the addition of an equal volume of 2X Laemmli loading buffer.

### Sample preparation and Mass Spectrometry analysis

The purified LegK4 and LegK4^1–445^ proteins were subjected to *in vitro* phosphorylation with non-radioactive ATP, as described in the phosphorylation assays section, prior to SDS-PAGE separation and mass spectrometry analysis. The separated proteins were subjected to in-gel reduction, carbamidomethylation and tryptic digestion. The phosphopeptides were isolated using TiO2 spin tips (Titansphere^TM^ Phos-TiO kit) according to the manufacturer protocol except for the elution steps. Retained phosphopeptides were eluted with 50 μL of 0.15 M ammoniumhydroxide solution then with another 50 μl of 60% CH_3_CN and 40% 0.3 M ammoniumhydroxide solution. Enriched phospho samples were analyzed by nano LC-MS/MS using an Ultimate nanoLC (ThermoFisherScientific) and a nanoESI-QToF-MS/MS mass spectrometry system (Q-Star XL, Applied Biosystems). Peptide separation was performed on a C18 PepMap micro-precolumn (Dionex) and a C18 PepMap nanocolumn (Dionex) where solvent was 0.1% HCOOH in H_2_O/CH_3_CN (95/5) and a gradient of solvent B (0.1% HCOOH in H_2_O/CH_3_CN (20/80)) was applied. MS data were acquired using Analyst QS 1.1 software (Applied Biosystems). Screening for phosphorylated peptides was achieved with ProteinPilot 4.0 software (Applied Biosystems) using the Paragon database search algorithm against a home-made database including the sequence of LegK4 and LegK4^1–445^.

## Additional Information

**How to cite this article**: Flayhan, A. *et al.* The structure of *Legionella pneumophila* LegK4 type four secretion system (T4SS) effector reveals a novel dimeric eukaryotic-like kinase. *Sci. Rep.* **5**, 14602; doi: 10.1038/srep14602 (2015).

## Supplementary Material

Supplementary Information

## Figures and Tables

**Table 1:** Data collection and refinement statistics.

	**AMP-PNP (native)**	**APO (SeLegK4^1–445^)**
Wavelength (Å)	0.87260	0.979
Resolution range (Å)	40.35–2.49 (2.579–2.49)	44.69–3.706 (3.838–3.706)
Space group	P 1 21 1	P 3 2 1
Cell parameters a, b, c (Å) α, β, γ (deg)	82.68 76.87 86.44 90 98.19 90	154.82 154.82 87.95 90 90 120
Total reflections	143101 (12847)	145163 (14726)
Unique reflections	37547 (3570)	13215 (1316)
Multiplicity	3.8 (3.6)	11.0 (11.2)
Completeness (%)	99.42 (95.40)	100.00 (100.00)
Mean I/sigma(I)	11.14 (1.70)	13.79 (2.47)
Wilson B-factor	39.95	108.83
R-merge	0.1149 (0.8701)	0.239 (1.419)
R-meas	0.1338	0.2507
CC1/2	0.996 (0.6)	0.997 (0.694)
CC*	0.999 (0.866)	0.999 (0.905)
Refinement		
R-work	0.2037 (0.2718)	0.2821 (0.3557)
R-free	0.2577 (0.2997)	0.3107 (0.3621)
Macromolecules	6218	5596
Ligands	68	0
Water	192	0
Protein residues	778	697
RMS (bonds)	0.015	0.002
RMS (angles)	1.36	0.54
Ramachandran favoured (%)	94	95
Ramachandran allowed (%)	5.9	4.5
Ramachandran outliers (%)	0.1	0.46
Clashscore	11.37	8.42
Average B-factor	68.50	114.40
macromolecules	68.90	114.40
ligands	81.60	
solvent	49.50	

Statistics for the highest-resolution shell are shown in parentheses.

**Table 2:** Mass spectrometry identification of phosphorylated residues *in vitro*.

	**Sequence coverage (%)**	**Number of peptide identified**	**Phosphorylated peptide identified**	**Phosphorylated residue**
LegK4	95.5	184	VKR**pT**GDMGLFLSTPCGQTK	Thr823
LegK4^1–445^	84.2	192	LIKTP**pT**AK	Thr433
			TP**pT**AKMMAAIK	Thr433
			RFE**pS**DVVSR	Ser422
			RFESDVV**pS**R	Ser426
