# Erratum: The structure of *Legionella pneumophila* LegK4 type four secretion system (T4SS) effector reveals a novel dimeric eukaryotic-like kinase

**DOI:** 10.1038/srep20746

**Published:** 2016-02-03

**Authors:** Ali Flayhan, Célia Bergé, Nathalie Baïlo, Patricia Doublet, Richard Bayliss, Laurent Terradot

Scientific Reports 5: Article number: 1460210.1038/srep14602; published online: 09302016; updated: 02032016

In the original version of this Article, Laurent Terradot was incorrectly listed as being affiliated with ‘Department of Biochemistry, University of Leicester, Leicester LE1 9HN, United Kingdom’. The correct affiliation is listed below:

UMR 5086 BMSSI CNRS-Université de Lyon, Institut de Biologie et Chimie des Protéines, 7 Passage du Vercors, F-69367 Lyon Cedex 07, France

This error has now been corrected in the PDF and HTML versions of the Article.

