# Supplementary material for: The structure of Legionella pneumophila LegK4 type four secretion system (T4SS) effector reveals a novel dimeric eukaryotic-like kinase
Source: Sci Rep. 2015 Sep 30;5:14602. doi: 10.1038/srep14602 (PMC4588518; doi:10.1038/srep14602)
Supplement: Supplementary Information [file srep14602-s1.pdf]

Supplementary information

The structure of *Legionella pneumophila* LegK4 type four secretion system (T4SS) effector reveals a novel dimeric eukaryotic-like kinase

Ali Flayhan, Célia Bergé, Nathalie Baïlo, Patricia Doublet, Richard Bayliss, Laurent Terradot

Supplementary Figure S1-3

Figure legends

Figure S1

A) Electron density map (2Fo-Fc, blue) contoured at 1.3σ of AMP-PNP•LegK4$^{1\text{-}445}$ crystal structure. The model is shown as ball and sticks with carbon atoms colored in yellow, oxygen atoms in red and nitrogen atoms in blue. B) Fo-Fc omit map (green) for the AMP-PNP molecule of the crystal structure of AMP-PNP•LegK4$^{1\text{-}445}$ represented as in A). The model of AMP-PNP molecule is shown as ball and sticks with carbon atoms colored in magenta, oxygen in red and nitrogen in blue.

Figure S2

Structure-based sequence alignment of LegK1 (Uniprotkb Accession Number Q5WWA8), LegK2 (UAN Q5WUV1), LegK3 (UAN Q5WTP3) and LegK4 (UAN Q5WZW9) from *Legionnal pneumophila* strain Lens. For clarity, C-terminal sequences have been truncated. Secondary structure elements corresponding to LegK4$^{1\text{-}445}$ are indicated above the sequences. Active sites motifs are indicated below the sequences by colored bar and labeled accordingly. Identical residues are colored in white and shaded in red. Conserved residues are colored in red. Secondary structure predictions of LegK1-3 obtained using the server PSI-pred (http://bioinf.cs.ucl.ac.uk/psipred/) are indicated by blue (helices) or yellow (strands) bars on the sequences aligned with the cap and FHB domains.

Figure S3

Saxs data collection analysis and quality assessment. A) Kratky plot SAXS measurement of LegK4$^{1\text{-}445}$. The shape of the plot suggests that the protein is a mutidomain protein which shows limited flexibility. A P(r) plot for LegK4$^{1\text{-}445}$ with Dmax of 111 A ° (right) provides an excellent fit to the experimental data (left).

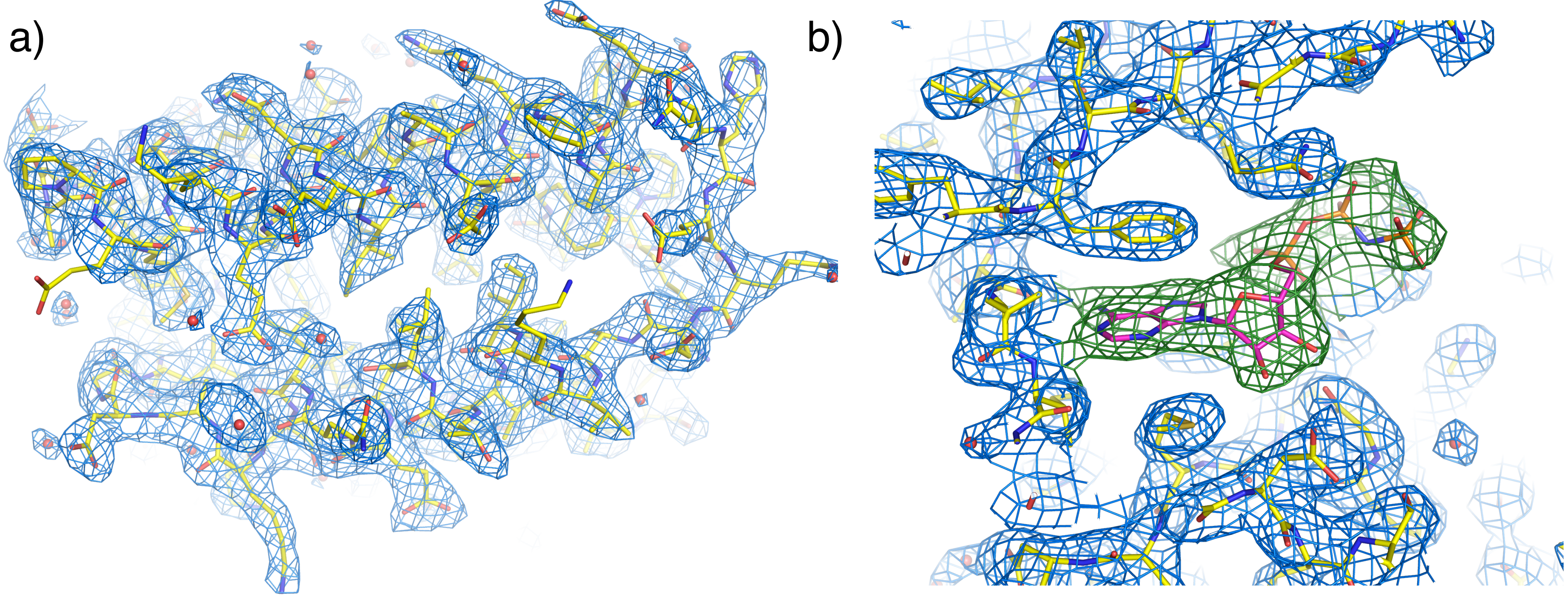

Fig. S1

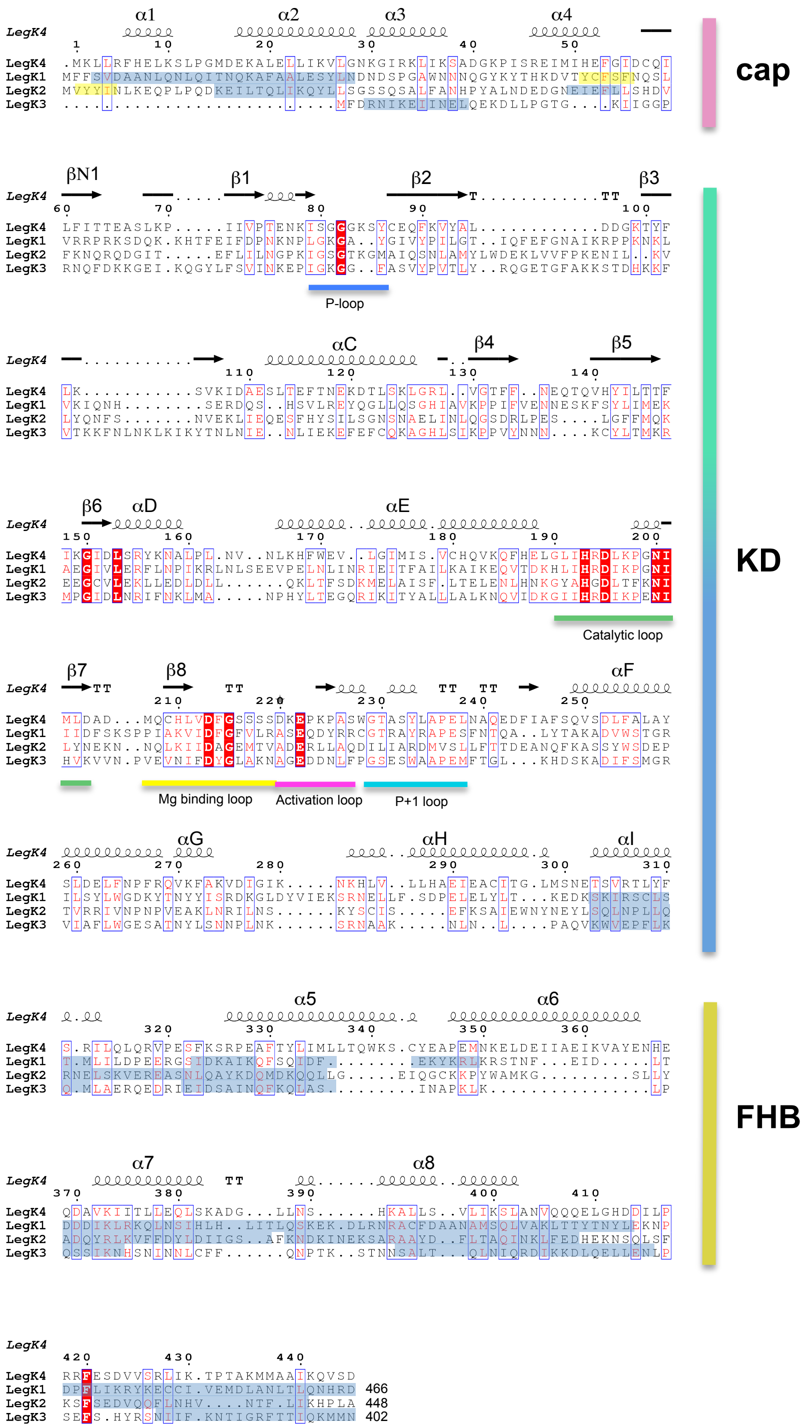
cap
KD
FHB
P-loop
Catalytic loop
Mg binding loop
Activation loop
P+1 loop
LegK4
LegK1
LegK2
LegK3

Fig. S2

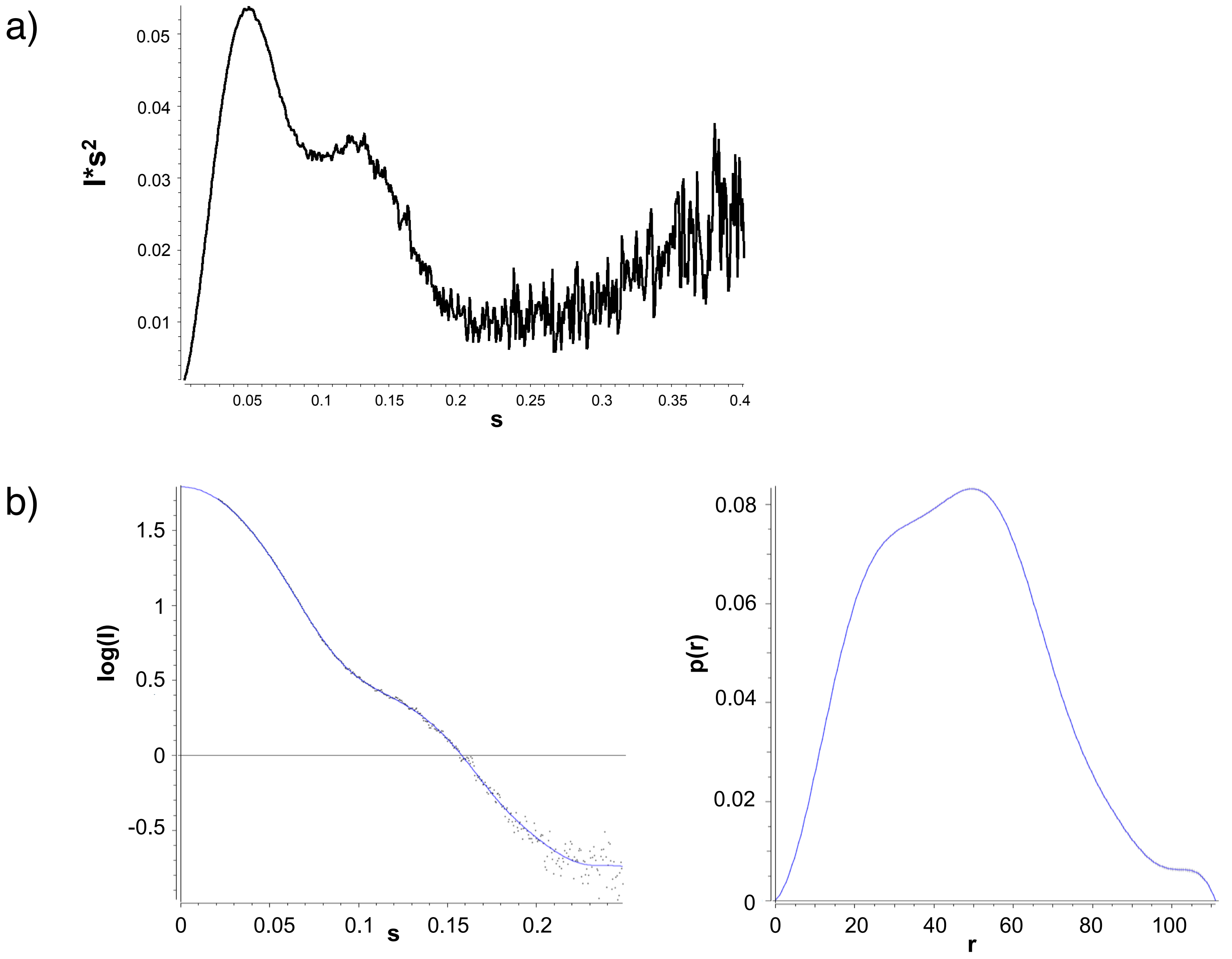
a)
I*s2
0.05
0.04
0.03
0.02
0.01
0.05
0.1
0.15
0.2
0.25
0.3
0.35
0.4
s
b)
log(I)
1.5
1
0.5
0
-0.5
0
0.05
0.1
0.15
0.2
s
p(r)
0.08
0.06
0.04
0.02
0
0
20
40
60
80
100
r

Fig. S3
